# Supplementary material for: Uncovering candidate genes involved in photosynthetic capacity using unexplored genetic variation in Spring Wheat
Source: Plant Biotechnol J. 2021 Feb 27;19(8):1537–52. doi: 10.1111/pbi.13568 (PMC8384606; doi:10.1111/pbi.13568)
Supplement: Supplementary file 3 — Table S7 Potential candidate genes identified for each association using Knetminer [file PBI-19-1537-s003.docx]

Supplementary Tables ST7- Potential candidate genes identified for each association using Knetminer

| **Description** | **Trait** | **Chrom** | **top snp ID** | **Pos** | **Pval** |  |  |
| --- | --- | --- | --- | --- | --- | --- | --- |
| Carotenoids reflectance 1 | **CRI1** | chr2A | chr2A-19048787 | 19048787 | 3.66E-10 | 2-25Mbp |  |

| **Gene** | **Name** | **Chr** | **Start** | **Evidence** |
| --- | --- | --- | --- | --- |
| TRAESCS2A02G012600 | UGT80A2 | 2A | 4949386 | Process: Regulation Of Photosynthesis Photoinhibition  Chloroplast Relocation  Photosystem II Stabilization |
| TRAESCS2A02G032500 | APS2 | 2A | 14808977 | Pathway: sulfate reduction II  Component: Chloroplast stroma  Process: Regulation Of Photosynthesis. Photoinhibition.  Chloroplast Relocation |
| TRAESCS2A02G053200 | FKBP19 | 2A | 21261065 | Process: Regulation Of Photosynthesis Photoinhibition  Chloroplast Relocation  Photosystem II Stabilization |
| TRAESCS2A02G036100 | PME1 | 2A | 15589802 | Process: Regulation Of Photosynthesis Photoinhibition  Chloroplast Relocation  Photosystem II Stabilization |
| TRAESCS2A02G010200 | CP5 | 2A | 3964835 | Process: Regulation Of Photosynthesis Photoinhibition  Chloroplast Relocation  Photosystem II Stabilization |

| **Description** | **Trait** | **Chrom** | **top snp ID** | **Pos** | **Pval** |  |  |
| --- | --- | --- | --- | --- | --- | --- | --- |
| Carotenoids reflectance 1 | **CRI1** | chr1B | chr1B-347845948 | 347845948 | 2.38E-07 |  |  |

No candidates identified

| **Description** | **Trait** | **Chrom** | **top snp ID** | **Pos** | **Pval** |  |  |
| --- | --- | --- | --- | --- | --- | --- | --- |
| Carotenoids reflectance 1 | **CRI1** | chr2B | chr2B-21973577 | 21973577 | 3.24E-05 |  |  |

No candidates identified

| **Description** | **Trait** | **Chrom** | **top snp ID** | **Pos** | **Pval** |  |  |
| --- | --- | --- | --- | --- | --- | --- | --- |
| Carotenoids reflectance 1 | **CRI1** | chr2D | chr2D-22432410 | 22432410 | 1.29E-08 |  |  |

No candidates identified

| **Description** | **Trait** | **Chrom** | **top snp ID** | **Pos** | **Pval** |  |  |
| --- | --- | --- | --- | --- | --- | --- | --- |
| Days to Anthesis | **DTA** | chr6B | chr6B-189683325 | 189683325 | 7.45E-07 | 16-19Mbp |  |

| **Gene** | **Name** | **Chr** | **Start** | **Evidence** |
| --- | --- | --- | --- | --- |
| TRAESCS6B02G031300 | BFRUCT3 | 6B | 18593033 | Trait: Flowering time trait |
| TRAESCS6B02G028300 | AGL86 | 6B | 16996335 | Trait: Flowering time trait |
| TRAESCS6B02G027900 | AGL36 | 6B | 16546325 | Trait: Flowering time trait |
| TRAESCS6B02G028100 | AGL92 | 6B | 16683849 | Trait: Flowering time trait |
| TRAESCS6B02G032400 | ATL79 | 6B | 18935971 | Trait: Flowering time trait |

| **Description** | **Trait** | **Chrom** | **top snp ID** | **Pos** | **Pval** |  |  |
| --- | --- | --- | --- | --- | --- | --- | --- |
| Days to Anthesis | **DTA** | chr5B | chr5B-27404243 | 27404243 | 8.12E-06 |  |  |

| **Gene** | **Name** | **Chr** | **Start** | **Evidence** |
| --- | --- | --- | --- | --- |
| TRAESCS5B02G027300 | RPS20B | 5B | 26445358 | Trait: Flowering time trait |
| TRAESCS5B02G029100 | BSL2 | 5B | 27830119 | Trait: Flowering time trait |
| TRAESCS5B02G028500 | UNE8 | 5B | 27506083 | Trait: Flowering time trait |
| TRAESCS5B02G029000 | KEA3 | 5B | 27820673 | Ontology: Flowering stage |
| TRAESCS5B02G028200 | IQD5 | 5B | 27294672 | Ontology: Flowering stage |
| TRAESCS5B02G028000 | - | 5B | 26950814 | Process: Photoperiodism, flowering |

| **Description** | **Trait** | **Chrom** | **top snp ID** | **Pos** | **Pval** |  |  |
| --- | --- | --- | --- | --- | --- | --- | --- |
| Enhanced vegetation index - greeness, biomass or chlorophyll | **EVI** | chr6B | chr6B-174740483 | 174740483 | 1.10E-06 | 17-19Mbp |  |

| **Gene** | **Name** | **Chr** | **Start** | **Evidence** |
| --- | --- | --- | --- | --- |
| TRAESCS6B02G032300 | PCMP-H22 | 6B | 18853707 | Process: Photosynthesis, Light Harvest, Chlorophyll Cycle, Regulation Of Chlorophyll, Chlorophyll Metabolic |
| TRAESCS6B02G028900 | TRAESCS6B02G028900 | 6B | 17414402 | Process: Photosynthesis, Light Harvest, Chlorophyll Cycle, Regulation Of Chlorophyll, Chlorophyll Metabolic |
| TRAESCS6B02G030900 | TRAESCS6B02G030900 | 6B | 18381079 | Process: Photosynthesis, Light Harvest, Chlorophyll Cycle, Regulation Of Chlorophyll, Chlorophyll Metabolic |

| **Description** | **Trait** | **Chrom** | **top snp ID** | **Pos** | **Pval** |  |  |
| --- | --- | --- | --- | --- | --- | --- | --- |
| Enhanced vegetation index - greeness, biomass or chlorophyll | **EVI** | chr3D | chr3D-523811772 | 523811772 | 1.50E-06 | 51-52Mbp |  |
|  |  |  |  |  |  |  |  |

| **Gene** | **Name** | **Chr** | **Start** | **Evidence** |
| --- | --- | --- | --- | --- |
| TRAESCS3D02G099500 | DREB2E | 3D | 51089961 | Trait: Chlorophyll content |
| TRAESCS3D02G099600 | MEE43 | 3D | 51108752 | Trait: leaf chlorosis |
| TRAESCS3D02G099400 | TRAESCS3D02G099400 | 3D | 51090218 | Process: Chlorophyll Catabolic Process |
| TRAESCS3D02G099800 | DPMS3 | 3D | 51686210 | Trait: Leaf chlorosis |

| **Description** | **Trait** | **Chrom** | **top snp ID** | **Pos** | **Pval** |  |  |
| --- | --- | --- | --- | --- | --- | --- | --- |
| Normalized difference vegetation index- green part of spectrum | **GNDVI** | chr2B | chr2B-153275048 | 153275048 | 7.03E-10 | 148-157Mbp |  |

| **Gene** | **Name** | **Chr** | **Start** | **Evidence** |
| --- | --- | --- | --- | --- |
| TRAESCS2B02G179200 | DMP10 | 2B | 153903431 | Component: Chloroplast  Trait: Leaf Chlorosis |
| TRAESCS2B02G177500 | CYP709B2 | 2B | 152568875 | Component: Chloroplast  Trait: Leaf Chlorosis |
| TRAESCS2B02G178400 | TRAESCS2B02G178400 | 2B | 153585725 | Process: Regulation of chlorophyll |
| TRAESCS2B02G178800 | TRAESCS2B02G178800 | 2B | 153854867 | Process: Regulation of chlorophyll |
| TRAESCS2B02G178000 | TRAESCS2B02G178000 | 2B | 153124400 | Process: Regulation of chlorophyll |
| TRAESCS2B02G177300 | CYP709B1 | 2B | 152472220 | Component: Chloroplast  Trait: Leaf Chlorosis |
| TRAESCS2B02G177600 | CYP709B1 | 2B | 152608315 | Component: Chloroplast  Trait: Leaf Chlorosis |
| TRAESCS2B02G177400 | CYP709B2 | 2B | 152503304 | Component: Chloroplast  Trait: Leaf Chlorosis |
| TRAESCS2B02G178700 | SKIP19 | 2B | 153759157 | Trait: Leaf Chlorosis |

| **Description** | **Trait** | **Chrom** | **top snp ID** | **Pos** | **Pval** |  |  |
| --- | --- | --- | --- | --- | --- | --- | --- |
| Normalized difference vegetation index- green part of spectrum | **GNDVI** | chr3B | chr3B-723108033 | 723108033 | 2.11E-06 |  |  |
|  |  |  |  |  |  |  |  |

| **Gene** | **Name** | **Chr** | **Start** | **Evidence** |
| --- | --- | --- | --- | --- |
| TRAESCS3B02G475000 | TRAESCS3B02G475000 | 3B | 723641665 | Process: Photosynthesis, Light Harvest, Chlorophyll Cycle, Regulation Of Chlorophyll, Chlorophyll Metabolic Processing |
| TRAESCS3B02G473000 | TRAESCS3B02G473000 | 3B | 722351335 | Process: Regulation of chlorophyll |
| TRAESCS3B02G473100 | TRAESCS3B02G473100 | 3B | 722359092 | Process: Regulation of chlorophyll |

| **Description** | **Trait** | **Chrom** | **top snp ID** | **Pos** | **Pval** |  |  |
| --- | --- | --- | --- | --- | --- | --- | --- |
| Leaf mass area overall | **LMAO** | chr3D | chr3D-336170196 | 336170196 | 3.29E-07 |  |  |

No candidates identified

| **Description** | **Trait** | **Chrom** | **top snp ID** | **Pos** | **Pval** |  |  |
| --- | --- | --- | --- | --- | --- | --- | --- |
| Leaf mass area overall | **LMAO** | chr1B | chr1B-65889406 | 65889406 | 5.71E-07 |  |  |
|  |  |  |  |  |  |  |  |

No candidates identified

| **Description** | **Trait** | **Chrom** | **top snp ID** | **Pos** | **Pval** |  |  |
| --- | --- | --- | --- | --- | --- | --- | --- |
| Normalized difference infrared index- canopy water content | **NDII** | chr6A | chr6A-497983531 | 497983531 | 6.33E-06 |  |  |

| **Gene** | **Name** | **Chr** | **Start** | **Evidence** |
| --- | --- | --- | --- | --- |
| TRAESCS6A02G271300 | CYP707A1 | 6A | 498006802 | Component: chloroplast  Enzyme: abscisic acid 8’-hydroxylase Process: Chloroplast RNA Processing, Cell Death, Release Of Seed From Dormancy, Growth, Regulation Of Transcription, Cellular Homeostasis, Response To Water Deprivation, Abscisic Acid Catabolic Process, Response To Auxin, Fruit Ripening |
| TRAESCS6A02G271900 | LPPE1 | 6A | 498946643 | MolFunc: Water Channel Activity  Process: Xylem Development, Root Development, Response To Water, Urea Transmembrane Transport, Response To Desiccation, Cellular Water Homeostasis, Water Transport, Stomatal Movement, Response To Water Deprivation |
| TRAESCS6A02G271800 | LPPE1 | 6A | 498808851 | MolFunc: Water Channel Activity  Process: Xylem Development, Root Development, Response To Water, Urea Transmembrane Transport, Response To Desiccation, Cellular Water Homeostasis, Water Transport, Stomatal Movement, Response To Water Deprivation |
| TRAESCS6A02G271600 | CCD4 | 6A | 498528029 | Pathway: abscisic acid biosynthesis  Process: Response To Water Deprivation |

| **Description** | **Trait** | **Chrom** | **top snp ID** | **Pos** | **Pval** |  |  |
| --- | --- | --- | --- | --- | --- | --- | --- |
|  | **NDMI** | chr2B | chr2B-669669845 | 669669845 | 2.73E-07 |  |  |

| **Gene** | **Name** | **Chr** | **Start** | **Evidence** |
| --- | --- | --- | --- | --- |
| TRAESCS2B02G425600 | PIP1-3 | 2B | 611282836 | Trait: drought tolerance, turgor pressure  Component: Apoplast, chloroplast stroma Process: Xylem Development, Seed Germination, Root Development, Response To Water Deprivation, Water Transport, Response To UV-B, Response To Desiccation, Cellular Water Homeostasis, Stomatal Movement |
| TRAESCS2B02G394900 | ARR4 | 2B | 559553430 | Component: Apoplast  Trait: water use efficiency, drought tolerance, photosynthetic ability, stomatal resistance  Process: Response To Blue Light, Seed Germination, Response To Far Red Light, Root Development, Response To Water Deprivation, Red Or Far-red Light Signaling, Response To Low Fluence Blue, Photomorphogenesis, Photosynthesis,Response To Light Stimulus, Response To UV-B, Detection Of Visible Light, Photosynthesis, Response To Red Light, Red Light Signaling Pathway, Transpiration |
| TRAESCS2B02G392900 | TCP7 | 2B | 557226759 | Component: Apoplast, chloroplast stroma  Trait: water use efficiency, drought tolerance, leaf relative water content, net photosynthetic rate, stomatal resistance  Process: Response To UV, Response To Blue Light, Seed Germination, Response To Far Red Light, Root Development, Response To Low Fluence Blue, Photomorphogenesis, Response To Light Stimulus, Response To UV-B, Cuticle Development, Flavonoid Glucuronidation, Cutin Biosynthetic Process, Response To Red Light, Photorespiration |
| TRAESCS2B02G396700 | PIP2-5 | 2B | 563043512 | Trait: drought tolerance, stomatal resistance Process: Xylem Development, Root Development, Urea Transport, Response To Water Deprivation, UV Protection, Water Transport, Red Or Far-red Light Signaling, Response To Water, Response To UV-B, Response To Desiccation, Photosynthesis, Cellular Water Homeostasis, Stomatal Movement |
| TRAESCS2B02G396800 | PIP2-1 | 2B | 563182221 | Trait: drought tolerance, stomatal resistance  Process: Xylem Development, Response To UV, Seed Germination, Root Development, Response To Water Deprivation, Water Transport, Response To Stress, Drought Recovery, Response To UV-B, Response To Desiccation, Cuticle Development, Flavonoid Glucuronidation, Photosynthesis, Cellular Water Homeostasis, Stomatal Movement |

| **Description** | **Trait** | **Chrom** | **top snp ID** | **Pos** | **Pval** |  |  |
| --- | --- | --- | --- | --- | --- | --- | --- |
| Normalized difference vegetation index | **NDVI** | chr7D | chr7D-608810464 | 608810464 | 2.56E-07 |  |  |

| **Gene** | **Name** | **Chr** | **Start** | **Evidence** |
| --- | --- | --- | --- | --- |
| TRAESCS7D02G501000 | KNAT3/KNOTTED1 | 7D | 607044614 | Process: Chlorophyll Metabolic Process, Positive Regulation Of hlorophyll Biosynthetic Process, Chlorophyll Cycle, Photosynthesis  Trait: Chlorophyll Content, Leaf Chlorosis, Delayed Senescence |

| **Description** | **Trait** | **Chrom** | **top snp ID** | **Pos** | **Pval** |  |  |
| --- | --- | --- | --- | --- | --- | --- | --- |
| Chlorophyll breakdown | **NPQI** | chr7A | chr7A-539405222 | 539405222 | 2.31E-07 |  |  |

| **Gene** | **Name** | **Chr** | **Start** | **Evidence** |
| --- | --- | --- | --- | --- |
| TRAESCS7A02G373800 | HY5 | 7A | 546702334 | Phenotype: Delayed Chlorophyll accumulation, Reduced Chlorophyll content  Trait: photosynthetic ability, leaf chlorophyll content  Process: Chlorophyll Biosynthetic Process, De-etiolation, Photoprotection, Photomorphogenesis, Photosynthesis |
| TRAESCS7A02G377600 | CIPK24 | 7A | 553036442 | Component: chloroplast stroma Process: Photosystem II Stabilization, Photosynthesis, Light Reaction, Photoprotection, Photosynthesis, Light Harvest, Photosynthetic NADP+ Reduction, Chloroplast Relocation, Photoinhibition |
| TRAESCS7A02G364400 | PP2A | 7A | 538689370 | Process: Leaf Senescence, Floral Organ Senescence |
| TRAESCS7A02G371100 | AtDBP1 | 7A | 544892406 | Trait: photosynthetic ability  Process: Photosynthesis, Photomorphogenesis, photorespiration |
| TRAESCS7A02G386300 | PIFI | 7A | 562001581 | Process: Chlorophyll Cycle, Chlorophyll Catabolic Process, Chlorophyll Metabolic Process, Chlorophyll Biosynthetic Process, Nonphotochemical Quenching |
| TRAESCS7A02G358600 | NDL3 | 7A | 531729875 | Component: chloroplast stroma  Process: Photosynthesis, photorespiration |
| TRAESCS7A02G383400 | BBX21 | 7A | 558402604 | Process: Regulation Of Chlorophyll Catabolic process, Photosynthesis, Photomorphogenesis, photoprotection |

| **Description** | **Trait** | **Chrom** | **top snp ID** | **Pos** | **Pval** |  |  |
| --- | --- | --- | --- | --- | --- | --- | --- |
| Plant senescence reflectance index | **PSRI** | chr3B | chr3B-718670251 | 718670251 | 3.43E-07 |  |  |

| **Gene** | **Name** | **Chr** | **Start** | **Evidence** |
| --- | --- | --- | --- | --- |
| TRAESCS3B02G470700 | TCP20 | 3B | 718849770 | Phenotype: Delayed Senescence  Process: Leaf Senescence, Positive Regulation of Leaf Senescence, photoprotection, photoinhibition, photosystem II stabilization |
| TRAESCS3B02G469000 | AHK3 | 3B | 715879545 | Phenotype: Delated Leaf Senescence, Dark Induced Senescence  Process: Photosynthesis |

| **Description** | **Trait** | **Chrom** | **top snp ID** | **Pos** | **Pval** |  |  |
| --- | --- | --- | --- | --- | --- | --- | --- |
| total chlorophyll content | **R750550** | chr2B | chr2B-2619111 | 2619111 | 9.55E-06 |  |  |

No candidates identified

| **Description** | **Trait** | **Chrom** | **top snp ID** | **Pos** | **Pval** |  |  |
| --- | --- | --- | --- | --- | --- | --- | --- |
| total chlorophyll content | **R750550** | chr2B | chr2B-153275048 | 153275048 | 6.29E-09 |  |  |
|  |  |  |  |  |  |  |  |

| **Gene** | **Name** | **Chr** | **Start** | **Evidence** |
| --- | --- | --- | --- | --- |
| TRAESCS2B02G177200 | ECB1/VAC1/PCMP-H73 | 2B | 152181989 | Process: Photosynthesis, photoprotection, photoinhibition, photosystem II stabilization |
| TRAESCS2B02G175300 | PDK | 2B | 150595802 | Process: Chlorophyll Catabolic Process Chlorophyll Biosynthetic Process Photosynthesis, Chloroplast relocation, Photosystem I Stabilization |
| TRAESCS2B02G178000 | TRAESCS2B02G178000 | 2B | 153903431 | Process: Chlorophyll Catabolic Process, Positive Regulation of Chlorophyll Catabolic Process |

| **Description** | **Trait** | **Chrom** | **top snp ID** | **Pos** | **Pval** |  |  |
| --- | --- | --- | --- | --- | --- | --- | --- |
| total chlorophyll content | **R750700** | chr3B | chr3B-736709296 | 736709296 | 2.72E-07 |  |  |
|  |  |  |  |  |  |  |  |

| **Gene** | **Name** | **Chr** | **Start** | **Evidence** |
| --- | --- | --- | --- | --- |
| TRAESCS3B02G477300 | PNSB5 | 3B | 725707259 | Process: Photosynthesis, photoprotection, photoinhibition, photosystem II stabilization |
| TRAESCS3B02G475900 | AK2 | 3B | 724750462 | Process: Photosynthesis, photoprotection, photoinhibition, photosystem II stabilization |
| TRAESCS3B02G490600 | GSTF12 | 3B | 736671865 | Process: Photosynthesis, photoprotection, photoinhibition, photosystem II stabilization |
| TRAESCS3B02G475000 | TRAESCS3B02G475000 | 3B | 723641665 | Process: Photosynthesis, photoprotection, photoinhibition, photosystem II stabilization |
| TRAESCS3B02G483900 | TRAESCS3B02G483900 | 3B | 730314626 | Process: Photosynthesis, photoprotection, photoinhibition, photosystem II stabilization |
| TRAESCS3B02G483700 | TRAESCS3B02G483700 | 3B | 730308539 | Process: Photosynthesis, photoprotection, photoinhibition, photosystem II stabilization |
| TRAESCS3B02G484000 | TRAESCS3B02G484000 | 3B | 730342135 | Process: Photosynthesis, photoprotection, photoinhibition, photosystem II stabilization |
| TRAESCS3B02G475800 | TRAESCS3B02G475800 | 3B | 724737433 | Process: Photosynthesis, photoprotection, photoinhibition, photosystem II stabilization |
| TRAESCS3B02G486700 | TRAESCS3B02G486700 | 3B | 733064475 | Process: Photosynthesis, photoprotection, photoinhibition, photosystem II stabilization |

| **Description** | **Trait** | **Chrom** | **top snp ID** | **Pos** | **Pval** |  |  |
| --- | --- | --- | --- | --- | --- | --- | --- |
| Chlorophyll a content | **RARSa** | chr2A | chr2A-16347452 | 16347452 | 7.35E-08 |  |  |

| **Gene** | **Name** | **Chr** | **Start** | **Evidence** |
| --- | --- | --- | --- | --- |
| TRAESCS2A02G043200 | Sweet4/5 | 2A | 17249740 | Phenotype: Reduced Chlorophyll Content |
| TRAESCS2A02G041500 | ERF1 | 2A | 16745853 | Phenotype: Reduced Chlorophyll a content |
| TRAESCS2A02G018000 | TCP1 | 2A | 8543341 | Process: Photomorphogenesis  Component: chloroplast stroma |
| TRAESCS2A02G032500 | HS1 | 2A | 15881908 | Process: Positive regulation of Chlorophyll Biosynthesis, Regulation of Chlorophyll Catabolism |

| **Description** | **Trait** | **Chrom** | **top snp ID** | **Pos** | **Pval** |  |  |
| --- | --- | --- | --- | --- | --- | --- | --- |
| Chlorophyll a content | **RARSa** | chr2B | chr2B-20043565 | 20043565 | 8.67E-07 |  |  |
|  |  |  |  |  |  |  |  |

| **Gene** | **Name** | **Chr** | **Start** | **Evidence** |
| --- | --- | --- | --- | --- |
| TRAESCS2B02G041600 | ABCB23 | 2B | 18938082 | Process: Photosynthesis, photoprotection, photoinhibition, photosystem II stabilization |
| TRAESCS2B02G042400 | TRAESCS2B02G042400 | 2B | 19375108 | Process: Photosynthesis, photoprotection, photoinhibition, photosystem II stabilization |
| TRAESCS2B02G035500 | TRAESCS2B02G035500 | 2B | 17039816 | Process: Photosynthesis, photoprotection, photoinhibition, photosystem II stabilization |
| TRAESCS2B02G038500 | TRAESCS2B02G038500 | 2B | 17835675 | Process: Photosynthesis, photoprotection, photoinhibition, photosystem II stabilization |
| TRAESCS2B02G041500 | TRAESCS2B02G041500 | 2B | 18924780 | Process: Photosynthesis, photoprotection, photoinhibition, photosystem II stabilization |
| TRAESCS2B02G036200 | TRAESCS2B02G036200 | 2B | 17237518 | Process: Photosynthesis, photoprotection, photoinhibition, photosystem II stabilization |
| TRAESCS2B02G041200 | ASMT | 2B | 18819336 | Process: Photosynthesis |

| **Description** | **Trait** | **Chrom** | **top snp ID** | **Pos** | **Pval** |  |  |
| --- | --- | --- | --- | --- | --- | --- | --- |
| Chlorophyll a content | **RARSa** | chr2B | chr2B-154465685 | 154465685 | 4.08E-08 |  |  |

| **Gene** | **Name** | **Chr** | **Start** | **Evidence** |
| --- | --- | --- | --- | --- |
| TRAESCS2B02G177200 | ECB1/VAC1/PCMP-H73 | 2B | 152181989 | Process: Photosynthesis, photoprotection, photoinhibition, photosystem II stabilization |
| TRAESCS2B02G175300 | PDK | 2B | 150595802 | Process: Chlorophyll Catabolic Process Chlorophyll Biosynthetic Process Photosynthesis, Chloroplast relocation, Photosystem I Stabilization |
| TRAESCS2B02G178000 | TRAESCS2B02G178000 | 2B | 153903431 | Process: Chlorophyll Catabolic Process, Positive Regulation of Chlorophyll Catabolic Process |

| **Description** | **Trait** | **Chrom** | **top snp ID** | **Pos** | **Pval** |  |  |
| --- | --- | --- | --- | --- | --- | --- | --- |
| Chlorophyll a content | **RARSa** | chr7B | chr7B-135775607 | 135775607 | 5.78E-06 |  |  |

| **Gene** | **Name** | **Chr** | **Start** | **Evidence** |
| --- | --- | --- | --- | --- |
| TRAESCS7B02G116700 | POT8 | 7B | 135578175 | Component: chloroplast  Process: Regulation of chlorophyll  Trait: leaf chlorosis, chlorophyll content |

| **Description** | **Trait** | **Chrom** | **top snp ID** | **Pos** | **Pval** |  |  |
| --- | --- | --- | --- | --- | --- | --- | --- |
| chlorophyll b content | **RARSb** | chr2B | chr2B-153898371 | 153898371 | 1.83E-06 |  |  |

| **Gene** | **Name** | **Chr** | **Start** | **Evidence** |
| --- | --- | --- | --- | --- |
| TRAESCS2B02G177200 | ECB1/VAC1/PCMP-H73 | 2B | 152181989 | Process: Photosynthesis, photoprotection, photoinhibition, photosystem II stabilization |
| TRAESCS2B02G175300 | PDK | 2B | 150595802 | Process: Chlorophyll Catabolic Process Chlorophyll Biosynthetic Process Photosynthesis, Chloroplast relocation, Photosystem I Stabilization |
| TRAESCS2B02G178000 | TRAESCS2B02G178000 | 2B | 153903431 | Process: Chlorophyll Catabolic Process, Positive Regulation of Chlorophyll Catabolic Process |

| **Description** | **Trait** | **Chrom** | **top snp ID** | **Pos** | **Pval** |  |  |
| --- | --- | --- | --- | --- | --- | --- | --- |
| chlorophyll b content | **RARSb** | chr3B | chr3B-20186940 | 20186940 | 1.27E-07 |  |  |

| **Gene** | **Name** | **Chr** | **Start** | **Evidence** |
| --- | --- | --- | --- | --- |
| TRAESCS3B02G039600 | TRAESCS3B02G039600 | 3B | 19247178 | Process: Photosynthesis, photoprotection, photoinhibition, photosystem II stabilization |
| TRAESCS3B02G038900 | TRAESCS3B02G038900 | 3B | 18773304 | Process: Photosynthesis, photoprotection, photoinhibition, photosystem II stabilization |
| TRAESCS3B02G040100 | RPM1 | 3B | 19394360 | Process: De-etiolation, Photosynthesis  Component: Chloroplast  Trait: leaf chlorosis |
| TRAESCS3B02G039100 | MYB62 | 3B | 18846378 | Trait: leaf chlorosis  Phenotype: increased wax |
| TRAESCS3B02G040000 | SYTF | 3B | 19386567 | Process: Photosynthesis  Trait: leaf chlorosis |
| TRAESCS3B02G040200 | JGL | 3B | 19734359 | Trait: leaf chlorosis |
| TRAESCS3B02G041900 | TRPA1 | 3B | 20840849 | Process: Photosynthesis  Component: Chloroplast |
| TRAESCS3B02G041100 | ALT3 | 3B | 20382504 | Component: Chloroplast  Trait: leaf chlorosis |
| TRAESCS3B02G039700 | TRAESCS3B02G039700 | 3B | 19257562 | Process: regulation of chlorophyll |
| TRAESCS3B02G040300 | TRAESCS3B02G040300 | 3B | 19869669 | Process: regulation of chlorophyll |

| **Description** | **Trait** | **Chrom** | **top snp ID** | **Pos** | **Pval** |  |  |
| --- | --- | --- | --- | --- | --- | --- | --- |
| chlorophyll b content | **RARSb** | chr3B | chr3B-715184359 | 715184359 | 9.06E-07 |  |  |

| **Gene** | **Name** | **Chr** | **Start** | **Evidence** |
| --- | --- | --- | --- | --- |
| TRAESCS3B02G468900 | TROL | 3B | 714620182 | Trait: leaf chlorosis, chlorophyll content  Component: Chloroplast  Process: Photosynthesis, photoprotection, photoinhibition, photosystem II stabilization |
| TRAESCS3B02G470700 | TCP20 | 3B | 718849770 | Component: Chloroplast  Trait: leaf chlorosis, chlorophyll content  Process: Photomorphogenesis, photosynthesis, regulation of chlorophyll |
| TRAESCS3B02G477300 | PNSB5 | 3B | 725707259 | Component: Chloroplast  Process: Photosynthesis, photoprotection, photoinhibition, photosystem II stabilization |
| TRAESCS3B02G470400 | RPL27 | 3B | 718686777 | Component: Chloroplast  Process: Photosynthesis, photoprotection, photoinhibition, photosystem II stabilization  Trait: leaf chlorosis |
| TRAESCS3B02G475900 | AK2 | 3B | 724750462 | Component: Chloroplast  Process: Photosynthesis, photoprotection, photoinhibition, photosystem II stabilization  Trait: leaf chlorosis |
| TRAESCS3B02G475000 | TRAESCS3B02G475000 | 3B | 723641665 | Process: Photosynthesis, photoprotection, photoinhibition, photosystem II stabilization |
| TRAESCS3B02G468500 | TRAESCS3B02G468500 | 3B | 713391428 | Process: Photosynthesis, photoprotection, photoinhibition, photosystem II stabilization |
| TRAESCS3B02G475800 | TRAESCS3B02G475800 | 3B | 724737433 | Process: Photosynthesis, photoprotection, photoinhibition, photosystem II stabilization |
| TRAESCS3B02G470200 | TRAESCS3B02G470200 | 3B | 718287023 | Process: Photosynthesis, photoprotection, photoinhibition, photosystem II stabilization |
| TRAESCS3B02G476000 | SCRM2 | 3B | 724926061 | Trait: leaf chlorosis, chlorophyll content |
| TRAESCS3B02G469000 | AHK3 | 3B | 715879545 | Process: Photosynthesis, regulation of chlorophyll  Trait: chlorophyll content |
| TRAESCS3B02G469200 | CBSCBSPB4 | 3B | 716145475 | Process: Photosynthesis, regulation of chlorophyll |
| TRAESCS3B02G472000 | DCI1 | 3B | 720265674 | Process: regulation of chlorophyll  Component: Chloroplast |
| TRAESCS3B02G473300 | UBC27 | 3B | 722475647 | Component: Chloroplast |
| TRAESCS3B02G469700 | TRAESCS3B02G469700 | 3B | 716709589 | Process: Photosynthesis, regulation of chlorophyll |
| TRAESCS3B02G472800 | TRAESCS3B02G472800 | 3B | 721493462 | Process: regulation of chlorophyll |
| TRAESCS3B02G469800 | rpl2-B | 3B | 717180611 | Component: Chloroplast, chloroplast stroma |
| TRAESCS3B02G479600 | MTN1 | 3B | 727474885 | Component: Chloroplast  Trait: leaf chlorosis |
| TRAESCS3B02G471600 | TRAESCS3B02G471600 | 3B | 720182529 | Process: regulation of chlorophyll |
| TRAESCS3B02G469600 | TRAESCS3B02G469600 | 3B | 716339106 | Process: regulation of chlorophyll |
| TRAESCS3B02G473000 | TRAESCS3B02G473000 | 3B | 722351335 | Process: regulation of chlorophyll |
| TRAESCS3B02G470000 | TRAESCS3B02G470000 | 3B | 717278885 | Process: regulation of chlorophyll |
| TRAESCS3B02G473100 | TRAESCS3B02G473100 | 3B | 722359092 | Process: regulation of chlorophyll |

| **Description** | **Trait** | **Chrom** | **top snp ID** | **Pos** | **Pval** |  |  |
| --- | --- | --- | --- | --- | --- | --- | --- |
| Carotenoid content | **RARSc** | chr3B | chr3B-20358957 | 20358957 | 1.25E-08 |  |  |

| **Gene** | **Name** | **Chr** | **Start** | **Evidence** |
| --- | --- | --- | --- | --- |
| TRAESCS3B02G040000 | SYTF | 3B | 19386567 | Process: Positive Regulation Of Carotenoid Biosynthetic Process |
| TRAESCS3B02G039600 | TRAESCS3B02G039600 | 3B | 19247178 | Process carotenoid Biosynthetic Process |
| TRAESCS3B02G040300 | TRAESCS3B02G040300 | 3B | 19869669 | Process: regulation of chlorophyll |
| TRAESCS3B02G040400 | RPS10 | 3B | 19876841 | Component: Chloroplast |

| **Description** | **Trait** | **Chrom** | **top snp ID** | **Pos** | **Pval** |  |  |
| --- | --- | --- | --- | --- | --- | --- | --- |
| Carotenoid content | **RARSc** | chr7A | chr7A-676592398 | 676592398 | 1.84E-06 |  |  |

| **Gene** | **Name** | **Chr** | **Start** | **Evidence** |
| --- | --- | --- | --- | --- |
| TRAESCS7A02G485300 | FKBP13 | 7A | 676102667 | Process: Response To Far Red Light,, photoprotection, photoinhibition, photosystem II stabilization  Component: Chloroplast  Trait: leaf chlorosis |

| **Description** | **Trait** | **Chrom** | **top snp ID** | **Pos** | **Pval** |  |  |
| --- | --- | --- | --- | --- | --- | --- | --- |
| Carotenoid content | **RARSc** | chr7B | chr7B-722589303 | 722589303 | 4.66E-06 |  |  |

No candidates identified

| **Description** | **Trait** | **Chrom** | **top snp ID** | **Pos** | **Pval** |  |  |
| --- | --- | --- | --- | --- | --- | --- | --- |
| Carotenoid content | **RARSc** | chr7D | chr7D-608810464 | 608810464 | 1.05E-06 |  |  |

| **Gene** | **Name** | **Chr** | **Start** | **Evidence** |
| --- | --- | --- | --- | --- |
| TRAESCS7D02G503400 | TRAESCS7D02G503400 | 7D | 608487530 | Process: Carotenoid catabolic process |
| TRAESCS7D02G502700 | TRAESCS7D02G502700 | 7D | 608197042 | Process: Photosynthesis, photoprotection, photoinhibition, photosystem II stabilization |
| TRAESCS7D02G502800 | RTL2 | 7D | 608203575 | Process: photomorphogenesis  Component: Chloroplast |
| TRAESCS7D02G503100 | TRAESCS7D02G503100 | 7D | 608289285 | Process: regulation of chlorophyll |

| **Description** | **Trait** | **Chrom** | **top snp ID** | **Pos** | **Pval** |  |  |
| --- | --- | --- | --- | --- | --- | --- | --- |
| Respiration rate | **RDM** | chr1D | chr1D-397474458 | 397474458 | 1.27E-06 |  |  |

| **Gene** | **Name** | **Chr** | **Start** | **Evidence** |
| --- | --- | --- | --- | --- |
| TRAESCS1D02G298400 | MCM3 | 1D | 397072273 | Process: Photorespiration, photomorphogenesis |
| TRAESCS1D02G299200 | LECRKS6 | 1D | 397176249 | Process: Photorespiration, photomorphogenesis |
| TRAESCS1D02G298300 | TRAESCS1D02G298300 | 1D | 397067422 | Process: Photorespiration, photomorphogenesis |

| **Description** | **Trait** | **Chrom** | **top snp ID** | **Pos** | **Pval** |  |  |
| --- | --- | --- | --- | --- | --- | --- | --- |
| Normalized difference vegetation index- red part of spectrum | **RNDVI** | chr7A | chr7A-711982572 | 711982572 | 2.30E-06 |  |  |

No candidates identified

| **Description** | **Trait** | **Chrom** | **top snp ID** | **Pos** | **Pval** |  |  |
| --- | --- | --- | --- | --- | --- | --- | --- |
| Normalized difference vegetation index- red part of spectrum | **RNDVI** | chr7D | chr7D-608810464 | 608810464 | 9.92E-08 |  |  |

| **Gene** | **Name** | **Chr** | **Start** | **Evidence** |
| --- | --- | --- | --- | --- |
| TRAESCS7D02G501000 | KNAT3/KNOTTED1 | 7D | 607044614 | Process: Chlorophyll Metabolic Process, Positive Regulation Of Chlor, Chlorophyll Cycle, Photosynthesis  Trait: Chlorophyll Content, Leaf Chlorosis, Delayed Senescence |

| **Description** | **Trait** | **Chrom** | **top snp ID** | **Pos** | **Pval** |  |  |
| --- | --- | --- | --- | --- | --- | --- | --- |
| Structural independent pigment index, estimates Carotenoids:Chla ratio | **SIPI** | chr3B | chr3B-711119921 | 711119921 | 2.87E-06 |  |  |

| **Gene** | **Name** | **Chr** | **Start** | **Evidence** |
| --- | --- | --- | --- | --- |
| TRAESCS3B02G459900 | SUMO1 | 3B | 703330509 | Function: chlorophyll binding  Process: Photomorphogenesis, photosynthesis, photorespiration  Component: Chloroplast, Photosystem II, chloroplast stroma, Photosystem I reaction center  Trait: leaf chlorosis, chlorophyll content, chlorophyll ratio |
| TRAESCS3B02G452200 | ABI3 | 3B | 693338347 | Process: Chlorophyll biosynthetic process  Trait: leaf chlorosis, chlorophyll content |
| TRAESCS3B02G457100 | RPT5B | 3B | 698421194 | Component: Chloroplast, chloroplast stroma,  Process: Photomorphogenesis, photosynthesis, photorespiration, photoinhibition  Trait: leaf chlorosis, chlorophyll content |
| TRAESCS3B02G467700 | TOM20-1 | 3B | 710588678 | Component: Chloroplast, chloroplast stroma,  Process: Photomorphogenesis, photosynthesis, photorespiration, chlorophyll biosynthesis  Trait: leaf chlorosis, chlorophyll content |

| **Description** | **Trait** | **Chrom** | **top snp ID** | **Pos** | **Pval** |  |  |
| --- | --- | --- | --- | --- | --- | --- | --- |
| Structural independent pigment index, estimates Carotenoids:Chla ratio | **SIPI** | chr7D | chr7D-610551080 | 610551080 | 3.74E-06 |  |  |

| **Gene** | **Name** | **Chr** | **Start** | **Evidence** |
| --- | --- | --- | --- | --- |
| TRAESCS7D02G501000 | KNAT3/KNOTTED1 | 7D | 607044614 | Process: Chlorophyll Metabolic Process, Positive Regulation Of Chlorophyll Biosynthetic Process, Chlorophyll Cycle, Photosynthesis  Trait: Chlorophyll Content, Leaf Chlorosis, Delayed Senescence |
| TRAESCS7D02G503100 | TRAESCS7D02G503100 | 7D | 608289285 | Process: Regulation of Chlorophyll |

| **Description** | **Trait** | **Chrom** | **top snp ID** | **Pos** | **Pval** |  |  |
| --- | --- | --- | --- | --- | --- | --- | --- |
| chlorophyll content | **SPAD** | chr2B | chr2B-106930486 | 106930486 | 7.54E-06 |  |  |

| **Gene** | **Name** | **Chr** | **Start** | **Evidence** |
| --- | --- | --- | --- | --- |
| TRAESCS2B02G157600 | GUN5/CHLH | 2B | 131568447 | Component: Chloroplast, chloroplast stroma  Trait: leaf chlorosis, chlorophyll content  Process: Photosynthesis, photoprotection, photoinhibition, photosystem II stabilization |
| TRAESCS2B02G154700 | NTRC | 2B | 122741144 | Component: Chloroplast, chloroplast stroma  Trait: leaf chlorosis, chlorophyll content  Process: Photosynthesis, photoprotection, photoinhibition, photosystem II stabilization |
| TRAESCS2B02G163300 | DTX19 | 2B | 135691462 | Component: Chloroplast, chloroplast stroma  Trait: leaf chlorosis, chlorophyll content  Process: Photosynthesis, photoprotection, photoinhibition, photosystem II stabilization |
| TRAESCS2B02G154800 | FT | 2B | 122754604 | Component: Chloroplast  Process: Photosynthesis, Photomorphogenesis, positive regulation of chlorophyll  Trait: leaf chlorosis, chlorophyll content |
| TRAESCS2B02G144100 | VIPP1 | 2B | 110408776 | Component: Chloroplast, chloroplast stroma  Process: Photosynthesis, photosystem I stabilization, chloroplast relocation, chloroplast organization, de-etiolation, chlorophyll biosynthesis/catabolism |

| **Description** | **Trait** | **Chrom** | **top snp ID** | **Pos** | **Pval** |  |  |
| --- | --- | --- | --- | --- | --- | --- | --- |
| chlorophyll content | **SPAD** | chr2D | chr2D-16845994 | 16845994 | 1.96E-06 |  |  |

| **Gene** | **Name** | **Chr** | **Start** | **Evidence** |
| --- | --- | --- | --- | --- |
| TRAESCS2D02G043500 | CYP705A19 | 2D | 15628616 | Trait: leaf chlorosis  Component: Chloroplast |
| TRAESCS2D02G044200 | NPF2.11 | 2D | 15989772 | Trait: leaf chlorosis  Component: Chloroplast |
| TRAESCS2D02G044000 | NPF2.11 | 2D | 15933475 | Trait: leaf chlorosis  Component: Chloroplast |
| TRAESCS2D02G043400 | MRG1 | 2D | 15594940 | Trait: leaf chlorosis, chlorophyll content |
| TRAESCS2D02G043200 | TRAESCS2D02G043200 | 2D | 15583182 | Process: Photorespiration, photomorphogenesis |
| TRAESCS2D02G045400 | TRAESCS2D02G045400 | 2D | 16492957 | Process: Photorespiration, photomorphogenesis |
| TRAESCS2D02G045500 | TRAESCS2D02G045500 | 2D | 16494471 | Process: Photorespiration, photomorphogenesis |
| TRAESCS2D02G043300 | AFH3 | 2D | 15586444 | Component: Chloroplast stroma  Trait: leaf chlorosis |
| TRAESCS2D02G043800 | AT14A | 2D | 15920528 | Component: Chloroplast |
| TRAESCS2D02G044600 | TRAESCS2D02G044600 | 2D | 16128112 | Process: Photosynthesis |
| TRAESCS2D02G044400 | LTL1 | 2D | 16056955 | Component: Chloroplast |

| **Description** | **Trait** | **Chrom** | **top snp ID** | **Pos** | **Pval** |  |  |
| --- | --- | --- | --- | --- | --- | --- | --- |
| chlorophyll content | **SPAD** | chr5A | chr5A-3550988 | 3550988 | 4.72E-07 |  |  |

| **Gene** | **Name** | **Chr** | **Start** | **Evidence** |
| --- | --- | --- | --- | --- |
| TRAESCS5A02G004400 | NPF2.10 | 5A | 3085412 | Process: Photosynthesis, photoprotection, photoinhibition, photosystem II stabilization  Component: Chloroplast  Trait: leaf chlorosis |
| TRAESCS5A02G006100 | TRAESCS5A02G006100 | 5A | 3518743 | Process: Photosynthesis, photoprotection, photoinhibition, photosystem II stabilization |
| TRAESCS5A02G006200 | UBC17 | 5A | 3524460 | Process: Photosynthesis  Component: Chloroplast, chloroplast stroma  Trait: leaf chlorosis, chlorophyll content |
| TRAESCS5A02G004900 | CAS1 | 5A | 3281861 | Trait: leaf chlorosis |
| TRAESCS5A02G005900 | CAS1 | 5A | 3435395 | Trait: leaf chlorosis |
| TRAESCS5A02G004800 | 3BETAHSD/D2 | 5A | 3215100 | Trait: leaf chlorosis |
| TRAESCS5A02G005500 | ALY1 | 5A | 3390922 | Trait: leaf chlorosis |
| TRAESCS5A02G005800 | CYP94D1 | 5A | 3431398 | Component: Chloroplast |

| **Description** | **Trait** | **Chrom** | **top snp ID** | **Pos** | **Pval** |  |  |
| --- | --- | --- | --- | --- | --- | --- | --- |
| chlorophyll content | **SPAD** | chr6D | chr6D-456495062 | 456495062 | 8.69E-07 |  |  |

| **Gene** | **Name** | **Chr** | **Start** | **Evidence** |
| --- | --- | --- | --- | --- |
| TRAESCS6D02G378300 | IAA32 | 6D | 460732888 | Process: Photosynthesis, photoinhibition, photosystem II stabilization  Trait: chlorophyll b content, leaf chlorosis, chlorophyll content |
| TRAESCS6D02G370600 | AtNMNAT | 6D | 456495390 | Component: Chloroplast, chloroplast stroma  Process: Photosynthesis, photoprotection, photoinhibition, photosystem II stabilization |
| TRAESCS6D02G377900 | CYP97A3 | 6D | 460567567 | Component: Chloroplast  Path: lutein biosynthesis  Process: Photosynthesis, photoprotection, photoinhibition, photosystem II stabilization |
| TRAESCS6D02G378500 | PYRR | 6D | 460741027 | Pathway: flavin biosynthesis I  Component: Chloroplast  Process: Photosynthesis, photoprotection, photoinhibition, photosystem II stabilization |
| TRAESCS6D02G377000 | RAP2-11 | 6D | 459734435 | Process: Photomorphogenesis |

| **Description** | **Trait** | **Chrom** | **top snp ID** | **Pos** | **Pval** |  |  |
| --- | --- | --- | --- | --- | --- | --- | --- |
| water index 1 | **WI2** | chr3A | chr3A-616891767 | 616891767 | 9.04E-07 |  |  |

| **Gene** | **Name** | **Chr** | **Start** | **Evidence** |
| --- | --- | --- | --- | --- |
| TRAESCS3A02G372000 | ABI5 | 3A | 622718496 | Function: monooxygenase activity  Process: Seed germination, response to water deprivation, stomatal movement  Component: endomembrane system  Trait: water use efficiency, drought tolerance, turgor pressure, stomatal resistance |
| TRAESCS3A02G371900 | ABI5 | 3A | 622708130 | Function: monooxygenase activity  Process: Seed germination, response to water deprivation, stomatal movement  Component: endomembrane system  Trait: water use efficiency, drought tolerance, turgor pressure, stomatal resistance |
| TRAESCS3A02G371800 | ABI5 | 3A | 622277558 | Function: monooxygenase activity  Process: Seed germination, response to water deprivation, stomatal movement  Component: endomembrane system  Trait: water use efficiency, drought tolerance, turgor pressure, stomatal resistance |
| TRAESCS3A02G372200 | ABI5 | 3A | 622897640 | Function: monooxygenase activity  Process: Seed germination, response to water deprivation, stomatal movement  Component: endomembrane system  Trait: water use efficiency, drought tolerance, turgor pressure, stomatal resistance |
| TRAESCS3A02G372100 | ABI5 | 3A | 622816520 | Function: monooxygenase activity  Process: Seed germination, response to water deprivation, stomatal movement  Component: endomembrane system  Trait: water use efficiency, drought tolerance, turgor pressure, stomatal resistance |

| **Description** | **Trait** | **Chrom** | **top snp ID** | **Pos** | **Pval** |  |  |
| --- | --- | --- | --- | --- | --- | --- | --- |
| water index 2 | **WI3** | chr1B | chr1B-572799923 | 572799923 | 1.09E-07 |  |  |

| **Gene** | **Name** | **Chr** | **Start** | **Evidence** |
| --- | --- | --- | --- | --- |
| TRAESCS1B02G343500 | BZIP25 | 1B | 571707250 | Process: seed germination, response to water deprivation  Trait: water use efficiency, stomatal resistance, drought tolerance |
| TRAESCS1B02G342400 | COIL | 1B | 570515241 | Ontology: seed imbibition  Trait: drought tolerance |
| TRAESCS1B02G344100 | TRAESCS1B02G344100 | 1B | 572522811 | Ontology: seed imbibition  Process: xylem/root development, response to water, response to dessication, water transport, water homoeostasis, stomatal movement |
| TRAESCS1B02G343100 | BRCA1 | 1B | 571436630 | Ontology: seed imbibition  Process: xylem development, transpiration, seed germination, cuticle development |
| TRAESCS1B02G343800 | LIP1P | 1B | 572293070 | Ontology: seed imbibition  Process: xylem development, transpiration, seed germination, cuticle development |
| TRAESCS1B02G342100 | TRAESCS1B02G342100 | 1B | 570270312 | Ontology: seed imbibition  Process: xylem development, transpiration, seed germination, cuticle development |
| TRAESCS1B02G342000 | TRAESCS1B02G342000 | 1B | 570025130 | Ontology: seed imbibition  Process: xylem development, transpiration, seed germination, cuticle development |
| TRAESCS1B02G342700 | TRAESCS1B02G342700 | 1B | 571164481 | Trait: drought tolerance  Process: response to water deprivation, response to deep water |

| **Description** | **Trait** | **Chrom** | **top snp ID** | **Pos** | **Pval** |  |  |
| --- | --- | --- | --- | --- | --- | --- | --- |
| water index 2 | **WI3** | chr7A | chr7A-34727282 | 34727282 | 7.30E-06 |  |  |

| **Gene** | **Name** | **Chr** | **Start** | **Evidence** |
| --- | --- | --- | --- | --- |
| TRAESCS7A02G068300 | NAC066 | 7A | 34517336 | Ontology: seed imbibition  Process: xylem/root development, response to water deprivation, drought recovery, seed germination  Trait: water use efficiency, stomatal resistance, drought tolerance, leaf relative water content |
| TRAESCS7A02G068600 | TSM1 | 7A | 34721320 | Ontology: seed imbibition  Process: Photosynthesis, response to water deprivation, stomatal movement |
| TRAESCS7A02G068500 | CHLM | 7A | 34707264 | Ontology: seed imbibition  Process: Photosynthesis, response to water deprivation, stomatal movement |

| **Description** | **Trait** | **Chrom** | **top snp ID** | **Pos** | **Pval** |  |  |
| --- | --- | --- | --- | --- | --- | --- | --- |
| water index 4 | **WI4** | chr3A | chr3A-616891767 | 616891767 | 2.87E-08 |  |  |

| **Gene** | **Name** | **Chr** | **Start** | **Evidence** |
| --- | --- | --- | --- | --- |
| TRAESCS3A02G372000 | ABI5 | 3A | 622718496 | Function: monooxygenase activity  Process: Seed germination, response to water deprivation, stomatal movement  Component: endomembrane system  Trait: water use efficiency, drought tolerance, turgor pressure, stomatal resistance |
| TRAESCS3A02G371900 | ABI5 | 3A | 622708130 | Function: monooxygenase activity  Process: Seed germination, response to water deprivation, stomatal movement  Component: endomembrane system  Trait: water use efficiency, drought tolerance, turgor pressure, stomatal resistance |
| TRAESCS3A02G371800 | ABI5 | 3A | 622277558 | Function: monooxygenase activity  Process: Seed germination, response to water deprivation, stomatal movement  Component: endomembrane system  Trait: water use efficiency, drought tolerance, turgor pressure, stomatal resistance |
| TRAESCS3A02G372200 | ABI5 | 3A | 622897640 | Function: monooxygenase activity  Process: Seed germination, response to water deprivation, stomatal movement  Component: endomembrane system  Trait: water use efficiency, drought tolerance, turgor pressure, stomatal resistance |
| TRAESCS3A02G372100 | ABI5 | 3A | 622816520 | Function: monooxygenase activity  Process: Seed germination, response to water deprivation, stomatal movement  Component: endomembrane system  Trait: water use efficiency, drought tolerance, turgor pressure, stomatal resistance |

| **Description** | **Trait** | **Chrom** | **top snp ID** | **Pos** | **Pval** |  |  |
| --- | --- | --- | --- | --- | --- | --- | --- |
| water index 4 | **WI4** | chr3D | chr3D-611549913 | 611549913 | 1.24E-07 |  |  |

| **Gene** | **Name** | **Chr** | **Start** | **Evidence** |
| --- | --- | --- | --- | --- |
| TRAESCS3D02G543400 | ATSIZ1 | 3D | 613693809 | Ontology: seed imbibition  Process: seed germination, response to water, response to water deprivation  Trait: drought tolerance, stomatal resistance |
| TRAESCS3D02G540900 | TIP1-3 | 3D | 612272119 | Ontology: seed imbibition  Process: water transport  Function: water channel activity |
| TRAESCS3D02G543900 | TRAESCS3D02G543900 | 3D | 613739993 | Ontology: seed imbibition  Process: xylem/root development, response to water deprivation, water homeostasis, stomatal movement, water transport |
| TRAESCS3D02G542300 | TRAESCS3D02G542300 | 3D | 613383729 | Process: xylem/root development, response to water deprivation, water homeostasis, stomatal movement, |
| TRAESCS3D02G542600 | TRAESCS3D02G542600 | 3D | 613588996 | Process: xylem/root development, response to water deprivation, drought recovery, seed germination, water homeostasis, stomatal movement, cuticle development  Ontology: seed imbibition |
| TRAESCS3D02G541700 | ABCB6 | 3D | 612886488 | Process: xylem development, transpiration, seed germination, cuticle development  Ontology: seed imbibition |
| TRAESCS3D02G543100 | P58IPK | 3D | 613618087 | Process: xylem development, transpiration, seed germination, cuticle development |
